# Electrical Methods for Sensing Damage in Cement Mortar Beams Combined with Acoustic Emissions

**DOI:** 10.3390/ma15134682

**Published:** 2022-07-04

**Authors:** Andronikos Loukidis, Ilias Stavrakas, Dimos Triantis

**Affiliations:** Electronic Devices and Materials Laboratory, Department of Electrical & Electronics Engineering, University of West Attica, 250 Thivon Av., 122 44 Athens, Greece; a.loukidis@uniwa.gr (A.L.); ilias@uniwa.gr (I.S.)

**Keywords:** electrical resistivity, acoustic emissions, cement mortar, criticality

## Abstract

The temporal variation in terms of the “time-to-failure” parameter of the recordings of the electrical resistance and the acoustic emissions from concurrent measurements in three cement mortar specimens of prismatic shape that were subjected to a three-point bending test until fracture are studied. The novelty of the work at hand lies in the demonstration that the electrical resistance is described by a power law during the last stages of the loading protocols. The onset of the validity of the power law is indicative of the specimens’ imminent fracture, thus providing a useful pre-failure indicator. The above findings are supported by the analysis of the recorded acoustic signals in terms of the *F*-function and the *Ib*-value formulations.

## 1. Introduction

Understanding the fracture mechanisms and crack propagation processes when materials and structures are subjected to intense mechanical loading and the detection of the fracture region entrance constitutes a vital issue in recent engineering problems upon which current scientific research is focused. In this direction, many techniques have been developed based on the variation of the electrical properties as well as the recording and processing of acoustic signals [1,2,3] during the mechanical loading of materials until fracture. Regarding the study of electrical properties, we should mention the techniques of recording electromagnetic emissions [4,5] and weak electrical signals [6,7,8] as well as the variations in the electrical resistivity that highlight the limitation conditions of the durability of materials or the cause irreversible damages [9,10].

Mechanically loaded cement-based materials and structures have been studied in the past with the aid of electrical resistivity measurements to provide an early estimation regarding their mechanical characteristics, such as their durability [11] and pore structure [12], especially during their curing phase [13,14]. As cementitious materials age, their measured electrical resistance increases [15]. This can be attributed to the reduction in the moisture content in the cement pores, the closure of the intermediate pore space, and the initial cracking due to the low tensile strength of the early-age cementitious materials. Other factors that affect the electrical resistance are the composition of the cementitious materials (i.e., water/cement ratio, aggregates size, and porosity) [15] and their mechanical status [9,16]. Therefore, such measurements can provide real-time monitoring of the damage development and the crack propagation processes. In particular, the electrical resistance increases with the damage accumulation and decreases during healing processes [17]. Furthermore, the study of electrical resistance has been applied to fracture experiments in rocks [10,18], ceramic composites [19,20], and cement-based materials [21,22].

Another non-destructive testing (NDT) technique is the acoustic emissions (AE) technique, which is based on the detection of the transient mechanical waves produced due to the elastic energy bursts, released during the formation and propagation of cracks [23]. These waves travel within the volume of the material towards its surface in a spherical manner, where they are detected by properly attached piezoelectric sensors. Despite being introduced nearly 90 years ago [24,25,26], the AE technique is still under further development [27] and has gradually become a reliable NDT tool with successful applications both at the laboratory scale and in the field for the estimation of the remaining service life of full-scaled structures [28,29,30,31,32,33]. Several AE statistics, such as the rate of cumulative AE energy, the cumulative number of counts, the occurrence rate of AEs, and the distribution of the AE amplitudes, when properly analyzed, can provide useful findings regarding the internal crack evolution processes dominating the material before fracture and the overall response of the structures under mechanical load [34,35,36,37].

A popular evaluation method of the fracture evolution in materials is based on the variation of the so-called *b*-value, which describes the scaling of the AEs’ amplitude distribution through the Gutenberg–Richter law [37,38]. Several studies have been conducted for establishing a systematic change of the *b*-value during the different stages of a fracture process, aiming to use the *b*-value analysis as a tool for assessing fracture evolution processes [31,39,40]. Shiotani et al. [39] proposed an improved methodology for conducting the *b*-value analysis without sacrificing significantly the accuracy of the results, using the statistical parameters of the mean value and the standard deviation of the recorded AE amplitudes considered. The improved *b*-value (*Ib*-value) is a parameter derived from the AE’s amplitude’s distribution, and it is related to the impeding fracture, as its value changes during the fracturing process [39]. It is accepted that the temporal variation of the *Ib*-values is indicative of the system’s proximity to criticality (i.e., impending fracture) [39]. The *Ib*-value is calculated according to:

(1) $$Ib=\frac{\log_{10}N\left(\mu -a_{1}\sigma \right)-\log_{10}N\left(\mu +a_{2}\sigma \right)}{\left(a_{1}+a_{2}\right)\sigma }$$

with *μ* being the average value of the AE amplitude distribution of a group comprising *Ν* AE hits/events; *σ* being the standard deviation of the same group; $a_{1}$ is a constant related to the smaller AE amplitudes; $a_{2}$ is a constant related to the fracture level. In the literature, the values of the constants $a_{1}$ and $a_{2}$ lie between 1.0 and 2.0. Here, following the most common approach, both constants were set equal to 1.0.

The *F*-function [8,41] constitutes an alternative way to analyze the acoustic activity by exploiting the average frequency of the occurrence of the AE hits/events during a window of a specific number of consecutive AE hits/events. The *F*-function is calculated using *Ν* consecutive interevent times (time intervals between consecutive AE hits/events). The number (*Ν*) depends on the total number of the recorded AE hits/events during the experiment, with the minimum value of *Ν* being equal to 10. The respective value of the *F*-function is defined as the inverse of the mean value of the *Ν* consecutive interevent times. Each value of the *F*-function is associated with the average time value *τ* of the occurrence time instants of the consecutive AE hits/events, which are used to calculate the specific value of the *F*-function. In general, during the occurrence of the *k*th AE hit/event ($t_{k}$ time instant), the value of the *F*-function is calculated as the inverse of the average value of the $\left\{\delta t_{k},\delta t_{k-1},\ldots ,\delta t_{k-N+1}\right\}$ interevent times:

(2) $$F=\frac{{\left[\sum_{k-N+1}^{k}\left(\delta t_{i}\right)\right]}^{-1}}{N}$$

And it is associated to a time value *τ*, which is the average value of the $\left\{t_{k},t_{k-1},\ldots ,t_{k-N+1}\right\}$ time instants, since *N +* 1 consecutive AE hits/events are used for the calculation of *N* consecutive interevent times. Contrary to the common way of depicting the acoustic activity in terms of the “number of hits/events per second”, the temporal representation of the *F*-function has been proven advantageous, because it highlights with greater detail the variability of the acoustic activity, especially in the last stages prior to fracture. Since most of the acoustic activity usually occurs in the last seconds before fracture, the temporal representation of the *F*-function is usually shown in $\left(t_{f}-\tau \right)$ scale, where $t_{f}$ corresponds to the time instant of macroscopic fracture. This method of presentation and analysis of the recorded acoustic activity in fracture experiments has already been adopted by several researchers [42,43,44,45].

In a previous work [46], cement mortar specimens were subjected to consecutive loading and unloading stages of three-point bending until their fracture, while concurrent electrical resistance and AE recordings were conducted. The qualitative results in [46] and the present work show significant compatibility mainly on the fact that a steep increase in both the acoustic activity and the electrical resistance is recorded upon the specimens’ entrance to their fracturing region. In the work at hand, concurrent recordings of both the electrical resistance and the AEs when prismatic cement mortar specimens were subjected to a three-point bending (3PB) load are presented. The adopted herein loading protocol was quite different from the corresponding one in Reference [46], under the aspect of the mechanical behavior of the specimens. Specifically, the repeated mechanical loadings/unloadings may be used to study the possible memory effects at both electrical resistance and AE, but they cannot reveal information about the loading strength of a specimen or the remaining life of it. The variation of the electrical resistance was studied in the reverse time “time-to-failure” frame, focusing on the near failure region as well as in contrast to the AE activity. For the first time, the raw AE activity was also presented in terms of temporal variation and in juxtaposition to the corresponding load level. The comparative study of the temporal evolution of the electrical resistance and the acoustic activity in terms of the *Ib*-value and the *F*-function clearly show that they can provide pre-failure indication regarding the proximity to the impending mechanical failure.

## 2. Experimental Setup

**Figure 1:**
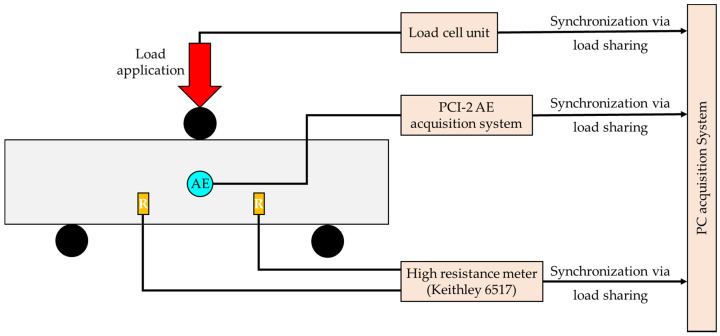
The experimental setup for the concurrent recordings of the electrical resistance and the AEs during the presented 3PB tests.

Three cement mortar specimens made from ordinary Portland cement subjected to 3PB load were tested. The specimens were of prismatic shape with dimensions 50 × 50 × 200 mm^3^. Details regarding their preparation can be found in Reference [46]. The specimens were placed on two solid metallic cylinders of 10 mm diameter, each one placed 85 mm from the specimens’ center. The load was applied at the central cross-section (see Figure 1) of the specimen via a third solid metallic cylinder. The experiments were conducted under quasi-static conditions with constant loading rates of 22 N/s for Exp-LR1 and Exp-LR2, while for Exp-HR it was 46 N/s. The loading system comprised an Instron 300 DX Static Hydraulic Testing Machine loading frame of 300 kN capacity and controlled by the Bluehill Universal software.

In order to record the electrical resistance, two gold-plated copper electrodes were implanted in the specimen during the preparation period ending in a thin (wire radius = 0.2 mm approximately) wire leading at the surface of the specimen in order for the high resistance meter to be connected. It is noted that the measured electrical resistance focused on the bulk region where the failure was expected to take place. Provided the size of the specimen and in order to avoid any impact on the mechanical behavior during the 3 PB tests, the sheet electrodes were orthogonal with dimensions of 4 × 3 mm^2^, while their thickness was on the order of 0.1 mm. It was expected during the 3 PB tests for the failure to take place in the central cross-section of the specimen and, specifically, initiate at the lower side of the specimen due to the excessive tensional stress. In order to be far from the expected failure plane and interact mechanically with the specimen, the electrodes were implanted parallel to the direction of the load and 40 mm from the cross-section (see Figure 1). The electrical resistance was measured using a sensitive high-resistance/low-current Keithley 6517 electrometer.

The PCI-2 AE acquisition system (Physical Acoustics Corp., West Windsor Township, NJ, USA) was used for the detection and recording of the AEs. One R6a sensor was properly attached in the center cross-section (see Figure 1) of each specimen using silicone as the coupling material, ensuring that most of the AE signals would be properly captured by the AE sensor. Note that both the electrical resistance and the AE techniques, initiated recording at the same time instant and were synchronized based on the applied load, which was continuously recorded not only by the internal system of the loading frame but also by both the electrical resistance and the AE measuring subsystems.

## 3. Results and Discussion

The presented experiments concerned three specimens of cement mortar of similar dimensions that were subjected to 3 PB until fracture. The experiments were divided into two categories depending on their loading rate. The first category included specimens loaded with a rate of 22 N/s (low-rate Exp-LR1 and Exp-LR2), while the specimens of the second category were subjected to 3 PB loading at a rate of 46 N/s (high-rate Exp-HR). The rate variation method was adopted to investigate whether the rate had an impact on the temporal evolution of electrical resistance and the generation rate of AEs or if a common behavior emerged independently from the loading rates.

**Figure 2:**
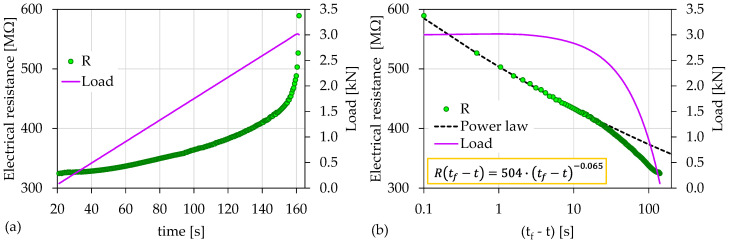
(**a**) The temporal evolution of the electrical resistance (*R*) in juxtaposition to the load applied for the experiment Exp-LR1; (**b**) the respective temporal evolution of the recorded electrical resistance (*R*) in terms of the “time-to-failure” parameter.

Figure 2a shows the temporal evolution of the recorded electrical resistance (*R*) in juxtaposition to the load applied for the case of the experiment Exp-LR1. The overall duration of this experiment was equal to approximately 141 s, and the maximum recorded value of the electrical resistance was 590 ΜΩ. Throughout the experiment, the electrical resistance (*R*) showed a constantly increasing trend, with a sharp increase occurring at the last stages of the experiment. In order to obtain a clearer view during these last stages, the temporal evolution of the electrical resistance (*R*) in juxtaposition to the load applied was plotted along a logarithmic scale against the “time-to-failure” parameter $\left(t_{f}-t\right)$, where $t_{f}$ is the time instant when the macroscopic fracture of the specimen occurred (Figure 2b). As can be seen, for $\left(t_{f}-t\right)>23\;s$ with the applied load attaining values greater than 2.6 kN, and until the end of the experiment, the evolution of the recorded electrical resistance data can be described by a power law of the form:

(3) $$R\left(t_{f}-t\right)=R_{1}\cdot {\left(t_{f}-t\right)}^{n}$$

with $R_{1}$ being a constant, and *n* is an exponent equal to 0.065.

The observed sharp increase in the recorded electrical resistance in Figure 2a, which corresponded to the aforementioned power law in Figure 2b, can be attributed to the intense crack development within the specimen, especially in the last loading stages before fracture. As the specimen entered its pre-failure region, a macrocrack network gradually formed around the area where the catastrophic fracture was expected to appear, which coincided with the area between the electrodes (i.e., the center of the specimen). These cracks interrupted the existing conductive paths of the material, resulting in an increase in the measured resistance. Thus, the increase in the number of cracks was accompanied by a consequent increase in the measured resistance.

**Figure 3:**
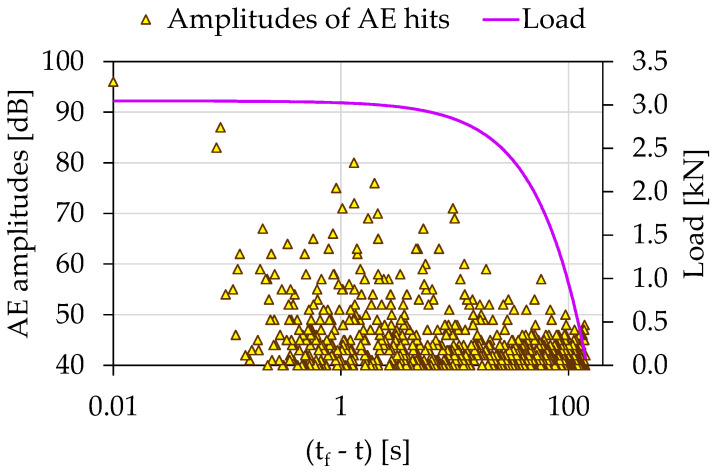
The temporal evolution of AE amplitudes in juxtaposition to the load applied for the experiment Exp-LR01 in terms of the “time-to-failure” parameter.

The temporal evolution of the amplitudes of the recorded AEs during the experiment Exp-LR1 in juxtaposition to the load applied is shown in Figure 3 in terms of the $\left(t_{f}-t\right)$ parameter. The total duration of the experiment was 141 s. Notice that the majority of the high amplitude AEs was recorded 10 s prior to the failure (i.e., for $\left(t_{f}-t\right)>10\;s$) and for load values greater than 2.8 kN.

**Figure 4:**
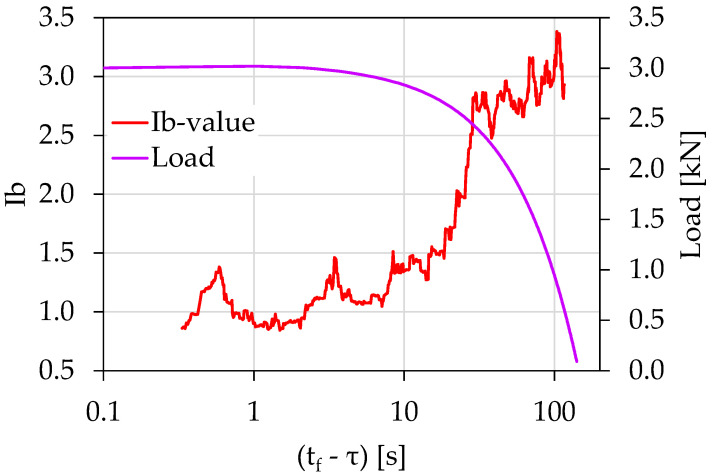
The temporal evolution of the *Ib*-value in juxtaposition to the load applied for the experiment Exp-LR1 in terms of the “time-to-failure” parameter.

Figure 4 shows the temporal evolution of the *Ib*-value calculated from the above-described AE data in juxtaposition to the load applied in terms of the $\left(t_{f}-\tau \right)$, with *τ* being the average value of the occurrence time of the AE hits, comprising the sliding “window” used for the calculation of the *Ib*-value. Each *Ib*-value was calculated using a sliding “window” of 70 consecutive AEs hits with a sliding step of 1 hit. Notice that the *Ib*-value initiated from relatively high values close to 3.5 and decreased progressively to the value of 2.7. At this point, a steep decrease in the *Ib*-value was observed from $\left(t_{f}-\tau \right)=30\;s$ until $\left(t_{f}-\tau \right)=14\;s$, with corresponding load values ranging between 2.4 and 2.7 kN. During this time frame, the *Ib*-value transitioned from 2.7 to 1.2. This behavior is indicative of the gradual coalescence of the microcrack network, which dominated the specimen, into a more extensive macrocrack network that eventually led to the catastrophic failure. This phenomenon could also be understood by studying Figure 3, where, as we moved closer to fracture, the recorded amplitude of the AEs increased. Later and until the end of the experiment, the *Ib*-value decreased further, attaining values close to the critical value of 1.0.

**Figure 5:**
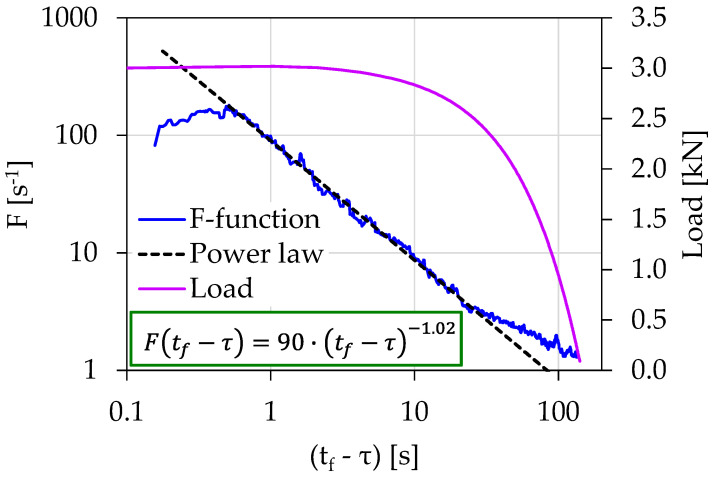
The temporal evolution of the *F*-function in juxtaposition to the load applied for the experiment Exp-LR1 in terms of the “time-to-failure” parameter.

Figure 5 shows the temporal evolution of the acoustic activity expressed through the *F*-function, in terms of the $\left(t_{f}-\tau \right)$. The *F*-function was calculated using the “sliding window” technique with a size of 20 consecutive AE hits. As can be seen from Figure 5, 24 s prior to fracture (i.e., for $\left(t_{f}-\tau \right)=24\;s$), the behaviors of the *F*-function can be described by a power law of the form:

(4) $$F\left(t_{f}-\tau \right)=A\cdot {\left(t_{f}-\tau \right)}^{m}$$

with A being a constant, and m is an exponent equal to 1.02. The validity of the power law extended from $\left(t_{f}-\tau \right)=25\;s$ until $\left(t_{f}-\tau \right)=0.5\;s$, with corresponding loading values raging between 2.6 and 3.01 kN. The existence of the power law could be attributed to the intense acoustic activity produced due to the activation of the fracture mechanisms during the specimen’s entrance to its pre-failure region.

**Figure 6:**
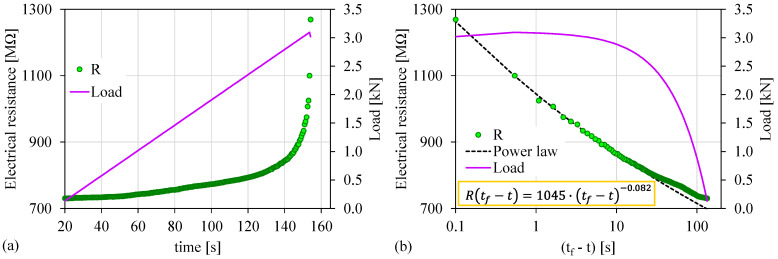
(**a**) The temporal evolution of the electrical resistance (*R*) in juxtaposition to the load applied for the experiment Exp-LR2; (**b**) the respective temporal evolution of the recorded electrical resistance (*R*) in terms of the “time-to-failure” parameter.

Figure 6a shows the temporal evolution of the recorded electrical resistance (*R*) in juxtaposition to the load applied for the case of the experiment Exp-LR2. The overall duration of this experiment was equal to approximately 135 s, and the maximum recorded value of the electrical resistance was 1270 ΜΩ. As can be seen, the electrical resistance showed an increasing trend throughout the experiment, which was intensified during the loading stages for *t* > 143 s. The corresponding temporal evolution of the electrical in juxtaposition with the load applied in $\left(t_{f}-t\right)$ scale is shown in Figure 6b. Notice that in a fashion similar to the experiment Exp-LR1, a power law of the form described in Equation (3) appears for $\left(t_{f}-t\right)>18\;s$, with a corresponding load value of 2.7 kN. The exponent *n* is now equal to 0.082.

The accumulated damage level seems to affect the total recorded electrical resistance of the specimens, as through the propagation of the cracks inside the specimens the conductive paths of the electric current are interrupted thus increasing the electrical resistance. In addition, it should be mentioned that the mechanical stimuli of the specimens were random due to the inhomogeneous nature of the cement mortar. The mechanical load was not the direct cause of the electrical resistance change. The electrical resistance changed due to the deformation that was caused by the loading of the specimen and the corresponding changes in the bulk structure. In addition, it is known that the mechanical load and the deformation were not linearly related to each other. Finally, the electrical resistance change was affected by several factors such as the free charge movement, lattice space, bulk discontinuities, crack paths, and crack path development. The novelty of the work at hand resides in the fact that by taking into account the random nature of the crack propagation within the specimens and the mechanical stimuli of the specimens to the constantly increasing mechanical load until fracture, this nonlinear increase in the recorded electrical resistance during the last seconds prior to failure was observed for all the examined specimens. Furthermore, this increase can be understood through a power law, the existence of which is indicative of the entry into the critical stages of various dynamic systems as was described in the influential work of Bak et al. (1987) [47]. In our case, the dynamic systems were the mechanically loaded specimens.

**Figure 7:**
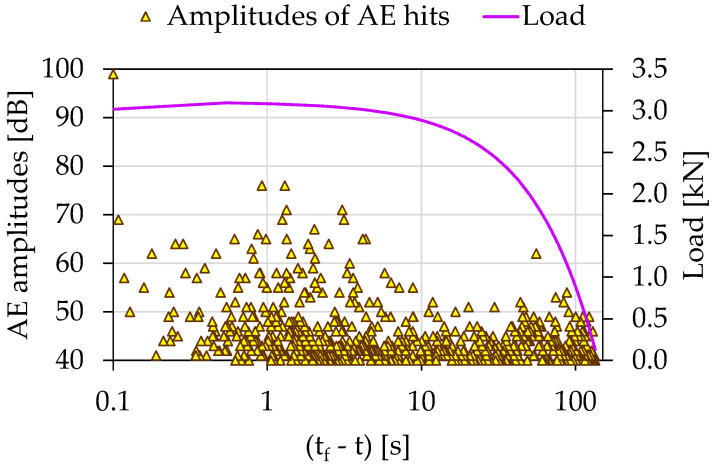
The temporal evolution of AE amplitudes in juxtaposition to the load applied for the experiment Exp-LR2 in terms of the “time-to-failure” parameter.

The temporal evolution of the recorded AE amplitudes in combination with the load applied for the experiment Exp-LR2 using the $\left(t_{f}-t\right)$ parameter is shown in Figure 7. The overall duration of this experiment was equal to approximately 135 s, while the majority of the highest AE amplitudes were recorded for $\left(t_{f}-\tau \right)>5\;s$. Therefore, to gain a clearer view during the last 5 s prior to fracture, it was decided, once again, to plot the temporal evolution of both the *Ib*-value and the *F*-function using the parameter $\left(t_{f}-\tau \right)$ along the logarithmic scale (in a similar procedure to experiment Exp-LR1).

In the initial loading stages, early microcracks (AE sources) were activated, producing a high number but of relatively low-amplitude AE hits. As the imposed mechanical load increased and the specimens’ load-bearing capacity decreased, the microcracks within the specimens were combined in a larger more extensive network of macrocracks, which produced fewer AE hits but of higher amplitude and duration. During the stabilization of the load in the last seconds before the collapse of the specimens, where the macroscopic crack was expected, the AE sources were excited almost in their entirety. The macroscopics fracture of the specimens was expressed as the last very AE hit with an amplitude of AE 99 dB. Conclusively, the initial high number of AE hits provided by a random crack network of the disorganized specimen system gradually handed over to a localized activity in the region of the expected fracture leading to an organized behavior leading to specimen’s failure.

**Figure 8:**
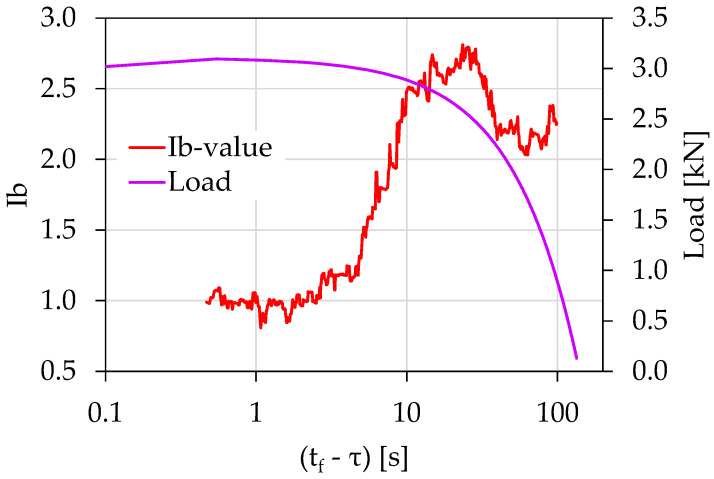
The temporal evolution of the *Ib*-value in juxtaposition to the load applied for the experiment Exp-LR02 in terms of the “time-to-failure” parameter.

Figure 8 shows the temporal evolution of the *Ib*-values in juxtaposition with the load applied for the case of the experiment Exp-LR2 using the $\left(t_{f}-\tau \right)$ parameter. In accordance with the experiment Exp-LR1, each *Ib*-value was calculated using a sliding “window” of 70 consecutive AEs hits and a sliding step of 1 hit. Notice that the *Ib*-value initiates from values close to 2.2, and after a brief fluctuation around those values, an increasing trend appeared, with the *Ib*-value approaching its highest value close to 2.8. Then, it followed a continuously decreasing trend which was intensified from $\left(t_{f}-\tau \right)=11\;s$ until $\left(t_{f}-\tau \right)=4\;s$, with corresponding load values ranging between 2.9 and 3 kN. During this time interval, the *Ib*-value fell from a value of 2.5 close to a value of 1.1. Finally, the *Ib*-values rested at the critical value 1.0 until the collapse of the specimen.

**Figure 9:**
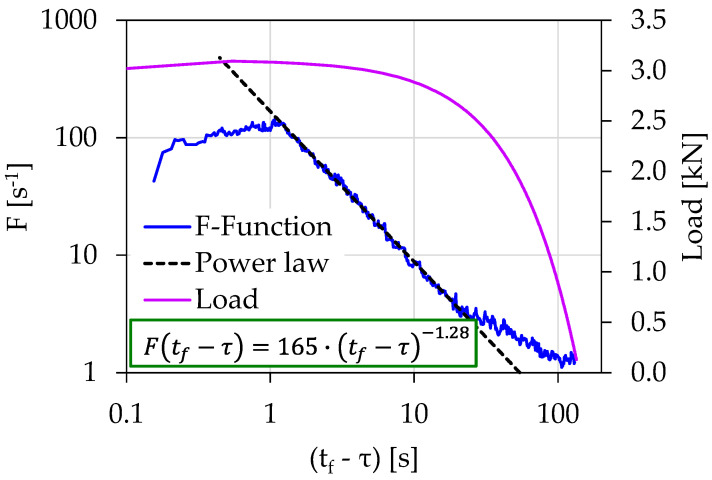
The temporal evolution of the *F*-function in juxtaposition to the load applied for the experiment Exp-LR2 in terms of the “time-to-failure” parameter.

Figure 9 shows the temporal evolution of the acoustic activity expressed through the *F*-function in terms of the $\left(t_{f}-\tau \right)$ for experiment Exp-LR2. The *F*-function in this case was calculated using the “sliding window” technique of 10 consecutive AE hits. As can be seen and in accordance with the findings of Exp-LR1, the *F*-function from $\left(t_{f}-\tau \right)=18.93\;s$ until $\left(t_{f}-\tau \right)=1.26\;s$ obeyed a power law in the form described by Equation (4), with the exponent *m* being equal to 1.28. The corresponding loading values ranged between 2.7 and 3.1 kN.

**Figure 10:**
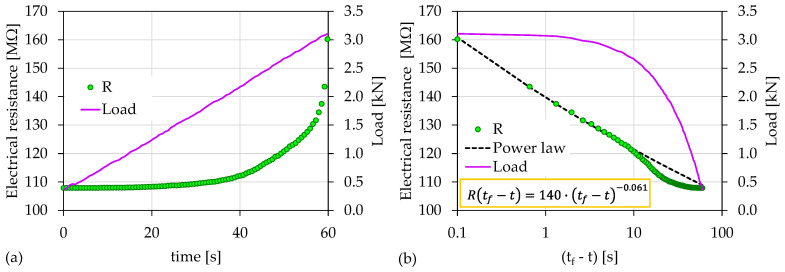
(**a**) The temporal evolution of the electrical resistance (*R*) in juxtaposition to the load applied for the experiment Exp-HR; (**b**) the respective temporal evolution of the recorded electrical resistance (*R*) in terms of the “time-to-failure” parameter.

Figure 10a shows the temporal evolution of the recorded electrical resistance (*R*) in juxtaposition to the load applied for the case of the experiment Exp-HR. The total duration of the experiment was 60 s, and the maximum recorded value of the electrical resistance was 160 ΜΩ. A similar behavior to the two previously presented experiments (i.e., Exp-LR1 and Exp-LR2) emerged, where the electrical resistance showed a continuously increasing trend that intensified during the last stages of the loading protocol for t>50 s. By depicting the temporal evolution of the recorded electrical resistance in terms of the $\left(t_{f}-t\right)$ scale, as shown in Figure 10b, a power law in the form described in Equation (3) appeared for $\left(t_{f}-t\right)>10\;s$, with a corresponding load value of 2.7 kN. The exponent *n* is equal to 0.061.

**Figure 11:**
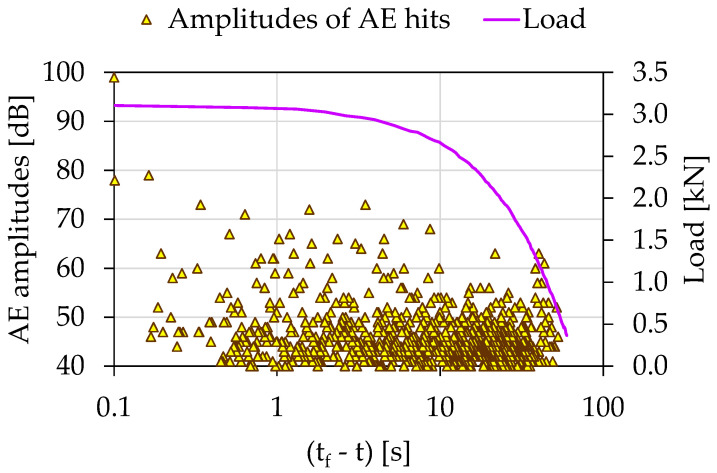
The temporal evolution of the AE amplitudes in juxtaposition to the load applied for the experiment Exp-HR in terms of the “time-to-failure” parameter.

The temporal evolution of the recorded AE amplitudes in juxtaposition to the load applied for the experiment Exp-HR using the $\left(t_{f}-t\right)$ parameter is shown in Figure 11. An early concentration of relatively high amplitude AEs can be observed for $36\;s<\left(t_{f}-t\right)<45\;s$. However, the majority of the recorded AEs with the highest amplitudes occurred during the last 10 s of the experiment. Following the same procedure as the two previously experiments (i.e., Exp-LR1 and Exp-LR2) the temporal evolution of both the *Ib*-values and the *F*-function was plotted using the time parameter $\left(t_{f}-\tau \right)$ along the logarithmic scale.

**Figure 12:**
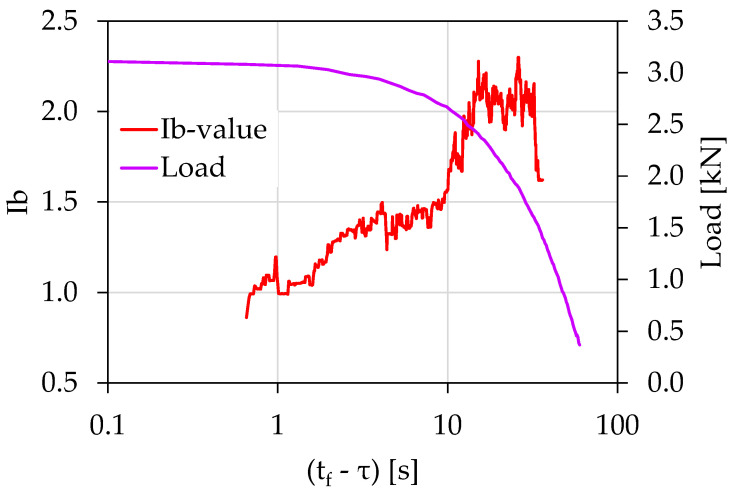
The temporal evolution of the *Ib*-value in juxtaposition to the load applied for the experiment Exp-HR in terms of the “time-to-failure” parameter.

Figure 12 shows the temporal evolution of the *Ib*-values in juxtaposition with the load applied for the case of the experiment Exp-HR in terms of the $\left(t_{f}-\tau \right)$ parameter. Here, each *Ib*-value was calculated using a sliding “window” of 100 consecutive AEs hits with a sliding step of 1 hit. The *Ib*-value initiated from a value equal to approximately 1.7 and almost immediately increased to values around 2.0. For approximately 20 s, the *Ib*-value fluctuated between 1.9 and 2.3. Then, a steep decrease was observed from $\left(t_{f}-\tau \right)=15\;s$ until $\left(t_{f}-\tau \right)=9\;s$, with corresponding load values ranging between 2.7 and 1.9 kN and the *Ib*-value transitioning from 2.3 to almost 1.3. At the last loading stages, the *Ib*-value continued to decrease further approaching a value close to 0.9.

**Figure 13:**
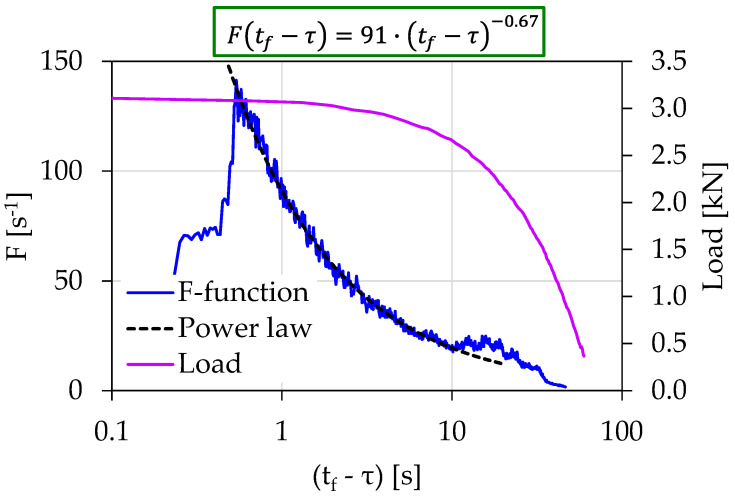
The temporal evolution of the *F*-function in juxtaposition to the load applied for the experiment Exp-HR in terms of the “time-to-failure” parameter.

The temporal evolution of the *F*-function in terms of the $\left(t_{f}-\tau \right)$ parameter for the Exp-HR experiment is shown in Figure 13. The *F*-function was calculated using the “sliding window” technique of 20 consecutive AE hits. In a similar fashion to the previously presented experiments, the *F*-function from $\left(t_{f}-\tau \right)=10\;s$ to $\left(t_{f}-\tau \right)=0.6\;s$ obeyed a power law in the form described by Equation (4), with the exponent *m* being equal to 0.67. The corresponding loading values ranged between 2.6 and 3.1 kN.

Table 1 summarizes the characteristics of the electrical resistance (*R*) power law, which is described by Equation (3), while Table 2 presents the corresponding characteristics of the *F*-function power law given by Equation (4). Both Table 1 and Table 2 present the time regions where the power laws extend, the values of exponents *n* and *m*, and the respective calculated values of the pre-exponential factors *R*_1_ and *A*. Additionally, the load rate and the fracture load, denoted as *L_f_* for each experiment, are presented. Each power law was correlated with the corresponding onset time instant in terms of the “time-to-failure” parameter and with the corresponding values of the normalized load applied (denoted as ℓ). The exponents *n* and *m* seemed to be indicators related to the intensity in the growth rate of the recorded resistance (*R*) and the acoustic activity (*F*-function). The values of the pre-exponential factors *R*_1_ and *A* corresponded, with a good approximation, to the value received by each power law, 1s before fracture. Finally, Table 3 presents the time instants when the calculated *Ib*-values crossed the value 1.5, signifying the approach of the specimens to the critical area close to the value of 1.0.

As can be observed, there was a temporal overlap between the validity time regions of the power laws, which characterized the temporal variation of both the electrical resistance (*R*) and the *F*-function. More specifically, for the Exp-LR1, the power laws started at approximately 24 s before fracture, with corresponding normalized load values at approximately ℓ ≈ 86%. For the Exp-LR2, the onset of the power laws started at approximately 20 s before fracture with corresponding load values equal to approximately ℓ ≈ 88%. For the case of Exp-HR, the power laws initiated at approximately 10 s before fracture, with corresponding load values around ℓ ≈ 87%.

**Figure 14:**
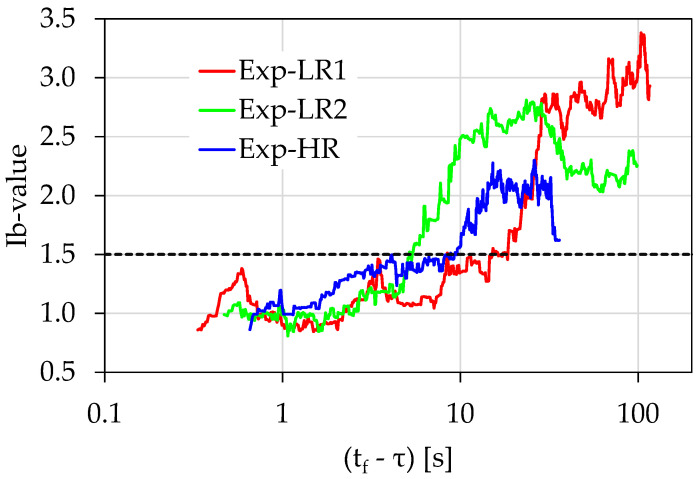
Comparative presentation of the temporal evolution of the *Ib*-values in juxtaposition to the load applied for the all the experiments in terms of the “time-to-failure” parameter.

In the case of the *Ib*-values, the situation was different: the higher load rate of the Exp-HR (46 N/s) seemed to affect the temporal evolution of the corresponding *Ib*-value, pushing it to cross the value 1.5 faster than the other experiments for $\left(t_{f}-t\right)<9\;s$, with a corresponding load value of approximately ℓ ≈ 88%. Contrary to this, for the case of the Exp-LR1 experiment, the *Ib*-value crossed 1.5 at $\left(t_{f}-t\right)<15\;s$, with a corresponding normalized load value ℓ ≈ 91% and for the experiment Exp-LR2 at $\left(t_{f}-t\right)<5.5\;s$, with a corresponding normalized load value ℓ ≈ 96% (Figure 14).

In the work at hand, it was shown, for the first time, that the electrical resistance shortly before the fracture of the specimens increased sharply by obeying a power law (see Equation (3)). Note that in the past [46], in the case of a similar specimen with a similar loading protocol and the same resistance measurement technique, a sharp increase in the electrical resistance was detected as the catastrophic fracture approached, albeit without the emergence of the law described by Equation (3). Clearly, further research is required, and experimental bending protocols should be applied to larger test specimens and extended to a wider range of materials.

It is noteworthy that the production rate of AE hits (expressed in terms of the *F*-function) in almost the same time period before the catastrophic fracture obeyed a similar power law (Equation (4)). Note that in a similar sample and with a similar loading protocol, the *F*-function and the electrical signal produced, known as pressure-stimulated currents (PSCs), began to obey a similar power law when the load reached 95% and 90% of the specimen’s total strength, respectively [8]. Furthermore, the loading rate seems to have no impact on the existence or the parameters of the power laws.

Recapitulating, based on the above experimental results, the electrical resistance can provide compatible and equivalent information to that of the acoustic activity (production rate of AE hits), as it can give information regarding the impending catastrophic fracture. The above findings are strongly supported, by the variation of the *Ib*-value, which is a well-known pre-failure index. It is indicative that the characteristic *Ib*-value drop steeply in the vicinity of 1.0, which characterizes the entrance to a pre-failure condition, coinciding with the power laws’ initiation time regions.

## 4. Conclusions

Cement mortar specimens were subjected to three-point bending loading up to the fracture using a load control protocol, and concurrent electrical resistance and acoustic emissions measurements were conducted. Two loading increase rates were selected in order to evaluate the impact of the rate on the experimental results.

The electrical resistance was measured using two gold-plated copper contact electrodes implanted during the specimen’s preparation in the region where the fracture was expected due to the adopted three-point bending loading. This region was symmetrically left and right from the mid-span of the specimen.

To elaborate on the acoustic emission data, the mean hit rate was calculated using the F-function, while the AE hit amplitudes were used to plot the temporal evolution of the *b*-value. In order to focus during the last stages of the loading protocol, all of the studied quantities were plotted as a function of the time to failure. The temporal variation of the electrical resistance was put in contrast to the acoustic emissions data in the vicinity of the fracture, and the results manifested a good correlation between them. In this work, for first time, it was revealed that the electrical resistance in the near-to-failure region can be described by a power law. The corresponding analysis of the AE arrival rate, presented in terms of the *F*-function, showed significant similarities with the behavior of the electrical resistance. Specifically, a power law also described the temporal behavior of the *F*-function in the vicinity of the fracture. It is worth mentioning that the loading rate seemed to have no impact on the existence or the factors of the power laws referred above.

The results revealed that the electrical resistance can provide compatible and equivalent information with the AE presented in terms of the *F*-function, actually signaling the imminent fracture. The above findings are strongly supported by the variation of a well-known pre-failure index: the *Ib*-value. It is indicative that the characteristic *Ib*-value drops steeply in the vicinity of 1.0, which characterizes the entrance to a pre-failure condition, coinciding to the power laws’ initiation time regions.

## Figures and Tables

**Table 1:** The details of the resistance (*R*) power law, $R\left(t_{f}-t\right)=R_{1}\cdot {\left(t_{f}-t\right)}^{n}$, for the presented experiments.

	Load Rate (N/s)	*L_f_* (kN)	*R*_1_ (MΩ)	*n*	(*t_f_* − *t*)	ℓ
Exp-LR1	22	3.01	504	0.065	<23 s	86%
Exp-LR2	22	3.09	1045	0.082	<18 s	88%
Exp-HR	46	3.11	140	0.061	<10 s	87%

**Table 2:** The details of the *F*-function power law, $F\left(t_{f}-\tau \right)=A\cdot {\left(t_{f}-\tau \right)}^{m}$, for the presented experiments.

	Load Rate (N/s)	*L_f_* (kN)	*A* (s^−1^)	*m*	(*t_f_* − *τ*)	ℓ
Exp-LR1	22	3.01	90	1.02	$0.5\;s<\left(t_{f}-\tau \right)<24\;s$	86%
Exp-LR2	22	3.09	165	1.28	$1.3\;s<\left(t_{f}-\tau \right)<20\;s$	88%
Exp-HR	46	3.11	91	0.67	$0.6\;s<\left(t_{f}-\tau \right)<10\;s$	87%

**Table 3:** The details of the *Ib*-values when the *Ib*-value < 1.5 for the presented experiment.

	Load Rate (N/s)	*L_f_* (kN)	(*t_f_* − *τ*)	ℓ
Exp-LR1	22	3.01	<15 s	91%
Exp-LR2	22	3.09	<5.5 s	96%
Exp-HR	46	3.11	<9 s	88%

## Data Availability

Not applicable.
